# SLA-printed nanographite-reinforced UV-curable resin composites for dental applications

**DOI:** 10.1038/s41598-026-62252-5

**Published:** 2026-07-13

**Authors:** Athulya Mullappally, Vishnu Vijay Kumar, G. R. Arpitha

**Affiliations:** 1 Manipal Institute of Technology Bengaluru, Manipal Academy of Higher Education, Manipal, India; 2 Structural Engineering, Division of Engineering, New York University Abu Dhabi (NYUAD), PO Box 129188, Abu Dhabi, United Arab Emirates

**Keywords:** Nanocomposite, Dental implants, Composite, Nanographite, SLA, Additive manufacturing, Engineering, Materials science

## Abstract

This study investigates stereolithography (SLA)-printed nanographite (NG)-reinforced UV-curable resin composites for dental applications. Anycubic plant-based resin was modified with 0, 0.5, 1, 3, and 5 wt% NG and evaluated for viscosity, UV absorbance, moisture content, water absorption, gel content, tensile, flexural, hardness, and microstructural behavior. The viscosity remained suitable for printing, decreasing slightly from 139 mPa·s for the control to 134 mPa·s at 5% NG. The 1% NG composite showed the best overall performance, with the highest tensile strength **(**20 MPa**)**, compared to 18 MPa for the control, and the highest flexural stress **(**50 MPa**)** and Shore D hardness (72). The control hardness was 70, while 5% NG dropped to 59. Gel content decreased from 99.86% (control) to 99.24% (5% NG), but higher loadings increased moisture and water uptake. Microscopy and SEM confirmed that 1% NG produced the most uniform morphology, whereas 3–5% NG showed agglomeration and defects. The results demonstrate that the controlled addition of NG enhances the mechanical performance of SLA-printed UV-curable resin composites, indicating their potential for provisional dental prosthetics applications.

## Introduction

Resin-based composites are pivotal for restorative procedures due to their aesthetic harmony with natural teeth, their support for minimally invasive techniques, and their suitability for direct application during dental appointments. Despite significant advances in their formulation over the years, these materials still pose mechanical challenges when used for extended periods in the oral environment. More common are fracture types, wear, and marginal degradation, which usually cause clinical failures and are produced in the back part of areas of high cyclic stress^1–3^. These shortcomings are inherent to the polymeric structure of the resin matrix, leading to brittleness, crack development, and volumetric contraction of the polymeric network during photopolymerization^4–6^. Attempts to solve these problems have focused on altering the filler stage and the filler-matrix relationship. The replacement of microfilled systems with nanofilled systems was a major development, resulting in greater load and polish at an acceptable viscosity^7^. Nanoscale fillers are also useful as reinforcement agents, to redistribute stress, limit the mobility of the polymer chain, and control the behaviour of crack propagation at the microscale^8,9^. The quality of dispersion and interfacial adhesion, however, is very dependent on their benefits. The stress may center in localities, even with minor agglomeration, which should be improved by it, and the opposite takes place^10,11^. Carbon-based nanomaterials have become of interest in this respect. A wide range of polymer-based systems that include graphene and its derivatives, such as graphene oxide and graphite nanoplatelets, have been reviewed with respect to their reinforcing properties, which are due to their high aspect ratio and inherent strength^12,13^. When incorporated into dental resins, these materials have demonstrated quantitative improvements in tensile and fracture strengths, as well as in microcrack propagation^14,15^. Their planar morphology enables efficient load distribution and optimal dispersion, and their surface chemistry can be tailored to make them compatible with the organic matrix^16^.

Nanographite (NG) is considered an efficient substitute, as it shares several of graphene’s mechanomedical strengths, yet is a more readily available material and more readily processed^17,18^. Its stratified framework promotes shear-induced alignment throughout mixing, which may provide anisotropic reinforcement of the polymer backbone^19^. Experimental studies have demonstrated that a low graphite weight fraction in graphite-reinforced polymers can significantly influence the mechanical response, particularly stiffness and crack growth resistance^14,20^. Its application in dentistry, however, is very limited, and the regularly examined concentration-dependent behavior of NG remains a poorly studied field. This will be especially noticeable when considering new fabrication approaches that impose new limitations on material formulation. Additive manufacturing has presented a new range of possibilities and challenges for dental materials. The concept of stereolithography (SLA) has been extensively adopted because of its competence in creating high-resolution structures with complicated forms directly based on computerized models^21^. It can accurately control layer thickness and surface finish through photopolymerization of liquid resins and, as such, has found applications in cases such as surgical guides, aligners, and provisional restorations^22,23^. The mechanical properties of standard SLA materials are generally not comparable to those required for the fabrication of definitive restorations^24,25^. The printed structures exhibit anisotropy, possess weak interlayer bonding, and demonstrate fracture resistance that is inferior to that of traditional composite processes^26,27^.

The nanofillers added to SLA-compatible resins increase their complexity. Compared with traditional composite fabrication, in which high filler loading can be achieved through mechanical mixing, SLA resins must be low-viscosity and optically transparent to allow natural light to penetrate and cure^28^. Nanoparticles may also scatter or absorb incident light, thereby decreasing the cure depth and leading to incomplete polymerization^29^. Experiments with silica nanoparticles and ceramic filler materials have also shown that small additions can enhance stiffness and strength; however, excessive loading reduces curing efficiency and dimensional accuracy^30,31^. Carbon-based fillers present several considerations in their application. Their high light-absorption properties can disrupt the photopolymerization kinetics of UV-reactive systems. Concurrently, their ability to form networks that percolate through the matrix facilitates multifunctional behavior, such as enhanced thermal stability and antimicrobial properties^32^. Achieving a homogeneous dispersion rate is crucial, as agglomerated particles can compromise structural integrity and lead to incomplete hardening^31^. Surface functionalization and controlled mixing have been explored to mitigate these issues, although their efficacy varies depending on the resin system and printing parameters employed^33,34^. Structurally, the interactions between nanofillers and the polymer matrix are pivotal in determining the mechanisms of composite failure. Crack initiation in resin-based systems typically occurs at points of weakness, such as the filler-matrix interface or unpolymerized regions. The incorporation of well-distributed NG can alter this behavior, promoting crack deflection, bridging, and pull-out, which are likely to enhance fracture resistance. Restoration materials are subjected not only to mechanical loading but also to thermal cycling, moisture exposure, and direct contact with biochemicals^35^. Over time, the resin matrix may weaken due to water sorption and hydrolytic degradation, and it may experience fatigue failure despite its initial strength from repeated loading^36^. The superior intrinsic properties of SLA-printed composites, along with stable filler-matrix interactions, are essential to enhance the resistance of composites to such conditions.

The present study examines the application and optimal parameters of NG-based resin in SLA printing in dentistry. The nanographitic content in the resin is varied at concentrations of 0.5%, 1%, 3%, and 5%, with corresponding changes being documented. The optimal nanorefinement is determined to maximize mechanical benefits without agglomeration, while maintaining printability and usability. Ultraviolet (UV) spectroscopy and viscosity measurements are conducted to ensure the printability of various combinations. Mechanical testing, including tensile, bending, and hardness assessments, is performed. Additionally, moisture content, water absorption, and gel content are estimated for different combinations. Optical and scanning electron microscopic characterization is conducted to analyze the microstructure. The study clearly identifies the optimal NG concentration for SLA printing, thereby bridging the gap between laboratory-scale nanocomposite development and potential clinical application. This research serves as a benchmark for the use of NG-based resin in SLA printing for dental applications.

## Materials and methods

### Materials

NG with a flake shape, a minimum ash content of 1.5%, a size of 0.1 µm, and 98% carbon content was used. The UV resin used for SLA printing is ANYCUBIC plant-based resin at 405 nm, with properties as shown in Table 1. The Anycubic plant-based UV-curable resin was selected as a model SLA photopolymer because of its stable printability, low viscosity, and consistent photopolymerization behavior under 405 nm UV irradiation. These characteristics make it suitable for systematically investigating the influence of nanographite reinforcement on the processing and mechanical performance of SLA-printed composites.

**Table 1:** Property of ANYCUBIC resin used for fabrication.

Property	Value
Elongation at break	14.20%
Hardness	79 D
Viscosity (25 °C)	150 mPa·s
Curing wavelength	405 nm
Liquid density	1.1 g/cm^3^
Solid density	1.184 g/cm^3^
Shrinkage	7.10%
Tensile strength	23.4 MPa
Curing time	6–10 s

### Fabrication

**Fig. 1:**
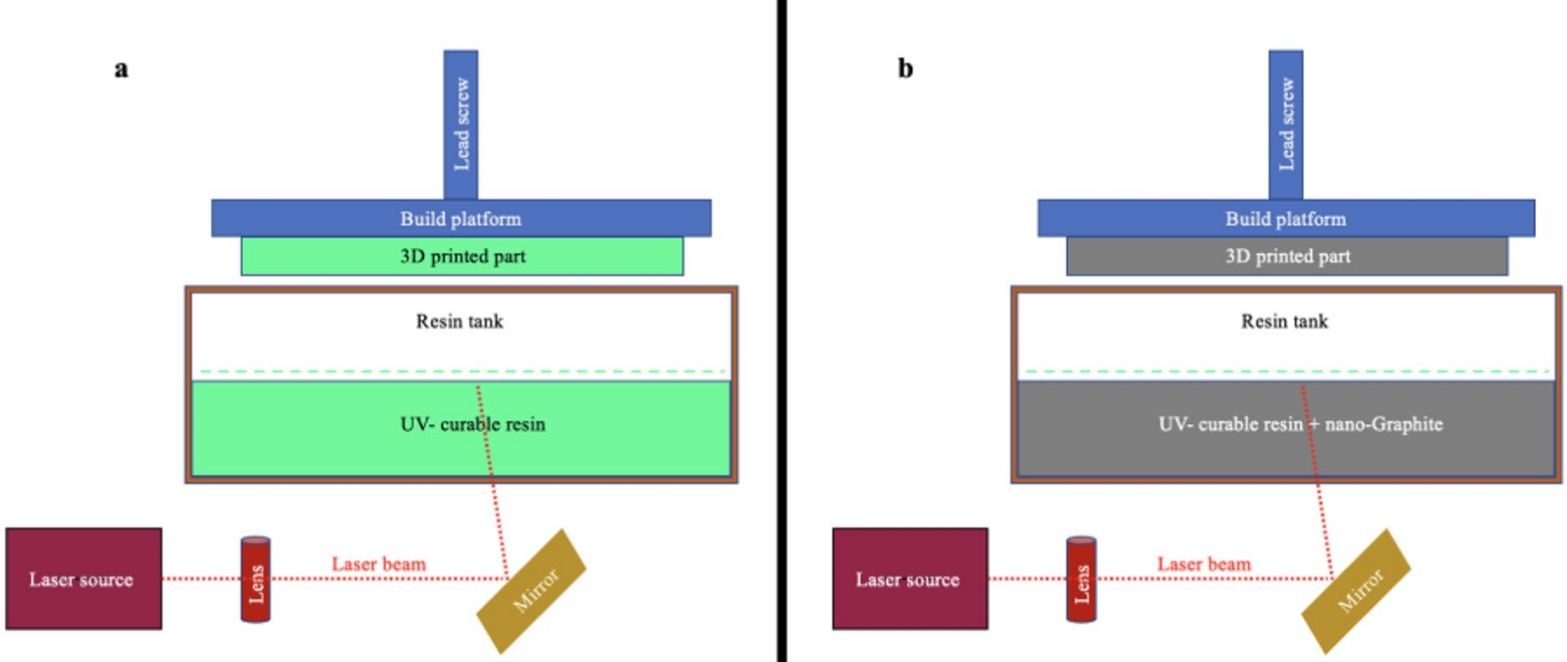
(**a**) SLA printing with resin (**b**) SLA printing with resin and NG.

The SLA additive manufacturing technique was used to fabricate the specimens. The Photon Mono Ultra 7 printer was employed, and the specific additive manufacturing parameters are detailed in Table 2. A plain UV-curable resin was fabricated and designated as the control specimen. Additionally, NG powder was incorporated into the resin at 0.5%, 1%, 3%, and 5% by weight (wt%). NG powder was dispersed in the UV-curable resin using a centrifuge mixer at 1500 rpm for 20 min, followed by ultrasonication at 40 kHz and 200W for 30 min to improve filler dispersion. The resin was maintained at approximately room temperature during mixing. Resin preparation was carried out in a dark environment to prevent premature photopolymerization. After mixing, the suspension was degassed under vacuum for 10 min, gently stirred immediately before printing to minimize particle sedimentation, and printed within 30 minutes of preparation. All specimens were printed with a horizontal build orientation. The layer-stacking direction corresponded to the printer’s Z-axis, and the raster pattern followed the default exposure strategy for the Photon Mono Ultra 7 SLA printer. Automatic support structures generated by the printer software were used as needed and removed after printing, prior to post-curing. Post-printing, the samples were immersed in isopropyl alcohol and then cured under UV light to ensure complete polymerization. The samples were then dried under ambient conditions. A total of five distinct sample types were fabricated for material testing and characterization, and the optimal percentage was investigated. Figure 1 depicts a schematic representation of the SLA printing with resin and the NG with UV curable resin.

**Table 2:** SLA printing parameters.

Property	Unit	Value
Layer thickness	mm	0.05
Bottom exposure time	s	30
Normal exposure time	s	2
Off time	s	0.5
Z lift distance	mm	6
Z lift speed	mm/s	4
Z retract speed	mm/s	6

### Viscosity measurement

The viscosity of the resin is a critical factor in additive manufacturing. The control and various NG-modified resins were evaluated for viscosity using a digital rotary viscometer. A consistent rotational speed of 40 rpm was maintained under ambient conditions. The viscosities of the different samples were analyzed and compared to assess their suitability for printing.

### UV spectroscopy

The efficiency of a resin for printing is determined by its absorption and transmittance properties. The resin must demonstrate adequate absorption at the curing wavelength to facilitate effective photopolymerization, while also allowing sufficient light penetration for proper layer formation. A GENESYS spectrometer is employed to measure these properties using UV spectroscopy. Both the control and various NG modifications are evaluated in the spectroscope to assess their characteristics.

### Moisture content

The moisture content in resin is a critical factor during SLA printing, as increased moisture can result in diminished material properties. To evaluate the moisture content in various samples, a moisture analyzer is employed to assess the total moisture content in both the control sample and different NG combinations. Initially, the various resins are placed in a crucible and subjected to heating at 130 °C for a continuous duration of 10 minutes in a closed environment. The weight of a known quantity of resin is measured before and after heating, and the total moisture loss during this process is analyzed to determine the moisture absorption characteristics of the resin.

1 $${\mathrm{Moisture}}\,{\mathrm{content}}\,\left( \% \right)\, = \frac{{{\mathrm{WR}}_{i} - {\mathrm{WR}}_{f} }}{{{\mathrm{WR}}_{i} }} \times 100$$

where ${\mathrm{W}\mathrm{R}}_{i}$ is the initial weight of resin, and ${\mathrm{W}\mathrm{R}}_{f}$ is the final weight of resin.

### Water absorption analysis

The extent of water absorption during use is a critical factor influencing the material’s applicability and long-term performance in dental applications. Water absorption was calculated in accordance with ASTM D570. The printed specimens, with nominal dimensions of 76 × 25 × 3 mm, were first dried in a hot-air oven at 50 °C for 24 h, then cooled to room temperature in a desiccator for 30 min and weighed to determine the initial dry mass. The conditioned specimens were then completely immersed in distilled water maintained at 25 °C for 24 h. After immersion, the specimens were removed, gently wiped with lint-free tissue to eliminate surface water, and immediately weighed to determine the wet mass. The specimen’s weight is measured before and after immersion, and the experiments are conducted under ambient conditions. The degree of water uptake determines the percentage of water absorbed, which helps assess the material’s susceptibility to hydrolytic degradation and swelling throughout its lifecycle.

2 $${\mathrm{Water}}\,{\mathrm{absorption}}\,\left( \% \right) = \frac{{W_{f} - W_{i} }}{{{\mathrm{WR}}_{i} }} \times 100$$

where: ${W}_{i}$ is the initial dry weight of the specimen before water immersion (g), ${W}_{f}$ is the final weight of the specimen after water immersion (g);

### Gel content measurement

The gel content in the SLA technique is instrumental for assessing the degree of crosslinking within the polymer network formed during photopolymerization. An increased gel content indicates greater crosslinking, enhancing the mechanical strength and durability of the specimen. The gel content in the SLA technique is determined by measuring the resin’s weight before and after solvent extraction. Subsequently, the gel content is calculated as the ratio of the insoluble fraction, typically expressed as a percentage:

3 $${\mathrm{Gel}}\,{\mathrm{content}}\,\left( \% \right) = \frac{{{\mathrm{WR}}_{i} n - {\mathrm{WR}}_{fi} }}{{{\mathrm{WR}}_{i} n }} \times 100$$

where ${\mathrm{W}\mathrm{R}}_{i}n$ is the initial weight of resin (g), and ${\mathrm{W}\mathrm{R}}_{fi}$ is the final weight of resin (g).

### Tensile test

The specimen was fabricated in accordance with ASTM D683, type 4, and the tensile properties of the various samples were analyzed using an Instron tensile testing machine. To ensure repeatability, three sets were conducted for each case. The samples were secured between the grips of the tensile testing machine, with one head stationary and the other movable. Tabs were applied to ensure proper gripping of the specimens and to prevent failure at the gripping area. The crosshead was moved at 5 mm/min, as per the standard, to analyze the tensile properties of the specimens. The tensile strength, modulus, and strain at break were computed at the specified strain rate and compared. Additionally, microscopic and microstructural analyses were performed on post-tensile specimens to examine the microscopic failure characteristics.

### Bending test

The specimens used to determine bending characteristics were fabricated in accordance with ASTM D790. A three-point bending test was conducted at a crosshead speed of 2 mm/min, with the specimen positioned between two roller supports and the crosshead applying force at the midpoint of the specimen. Three sets, each comprising control and NG variations, were tested to ensure repeatability. The flexural properties were calculated and subsequently compared.

### Hardness testing

The surface hardness of the specimen is very important for its use in dental applications. Shore D hardness testing was performed according to ASTM D-2240, with five specimens of each type. This testing provides essential data on the material’s resistance to surface deformation, which directly impacts its durability and performance in clinical settings.

### Microscopic and microstructural analysis

The post-tested specimens were examined using an optical DINOLITE AM4515T8 digital microscope, and microstructural characterization was performed using a QUANTA scanning electron microscope. Prior to SEM analysis, the specimen was sputter-coated with gold, and imaging was performed at various accelerating voltages.

## Results and discussions

### Fabrication

The composite was fabricated as previously described using the SLA additive manufacturing method. Control and NG reinforced composites with concentrations of 0.5, 1, 3, and 5 wt% were fabricated.

### Viscosity measurement results

The viscosity measurements were taken as discussed before for the control pure resin and the resin with NG additions at a constant torque in the viscometer. From the results, it can be seen that the pure resin showed a viscosity of 139 mPa.s, which corroborates earlier studies with the same resin^17^.

**Fig. 2:**
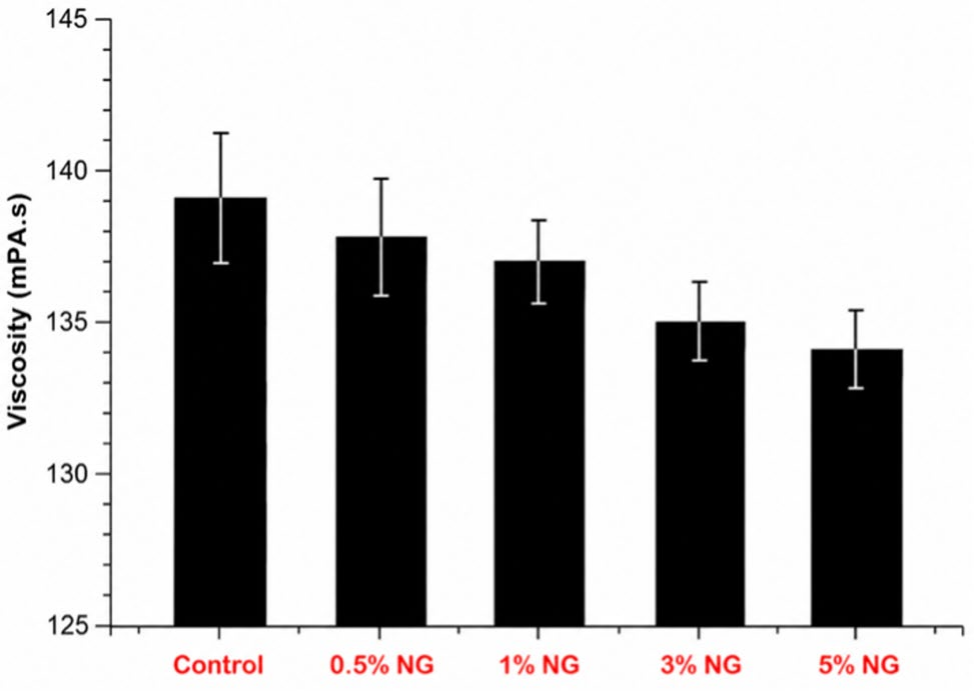
Viscosity measurements for different resins.

The 0.5%, 1%, 3%, and 5% showed viscosities of 138, 137, 135, and 134 mPa.s, respectively, as shown in Fig. 2. The slight decrease in viscosity with increasing NG concentration suggests improved flow characteristics of the resin-NG mixtures, which is beneficial for the SLA printing process. Lower viscosity facilitates smoother resin spreading and better layer formation during printing, potentially enhancing print resolution and surface finish. The viscosity values remain within a range suitable for SLA printing, indicating that the NG additions do not adversely affect the resin’s processability.

### UV Spectroscopy results

**Fig. 3:**
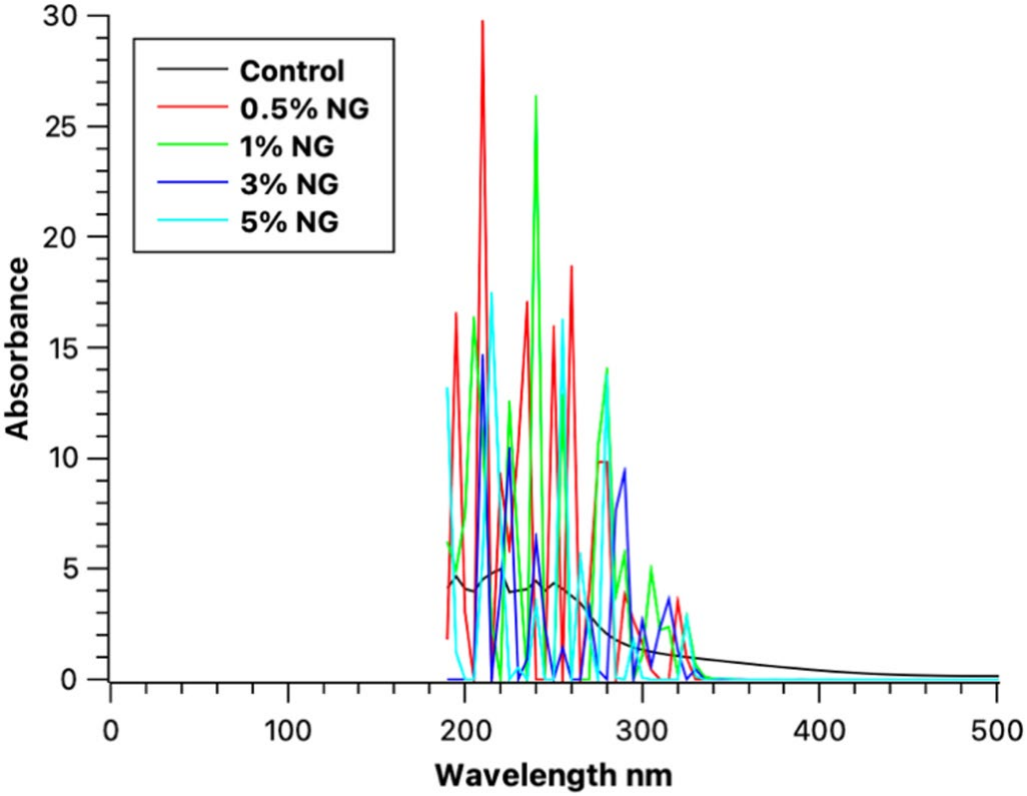
UV spectroscopy of the different resins used for printing.

The UV–visible absorbance spectra of the resin exhibit a strong correlation with NG loading, with pronounced changes in both peak intensity and spectral distribution in the 200–350 nm range. The control resin displays a relatively low, broad absorbance profile, with a maximum of approximately 3 absorbance units (AU) and a gradual decline beyond 300 nm. Upon the incorporation of NG, the spectra undergo substantial transformation, becoming more organized and intense. At a 0.5% NG concentration, sharp absorbance peaks emerge between 200 and 260 nm, with a maximum at 300 nm, indicating significant interaction with UV light. The addition of 1% NG maintains high absorbance levels, albeit with a slight reduction in peak intensity (2.5 AU), while still presenting several sharp transitions within the same wavelength region. However, with increased NG loading (3% and 5%), there is a noticeable decrease in peak intensity, with the highest absorbance diminishing to approximately 10 at 3% and further at 5%, accompanied by a loss in peak sharpness and increased spectral complexity, as shown in Fig. 3.

Such differences imply that low levels of NG increase UV absorption due to enhanced dispersion and greater photon-graphitic domain interaction, which is beneficial for photopolymerization activation during SLA processes. Higher NG loadings, in contrast, probably induce aggregation and light scattering, resulting in lower absorbance at the end and a lower penetration depth. High levels of NG can prevent access to photoinitiators or form optical opacities, reducing UV transmission. The findings suggest that the NG range (0.5–1%) is optimal to ensure high UV absorbance and reduce the negative impact of particle agglomeration and scattering as concentration increases.

### Moisture content results

**Fig. 4:**
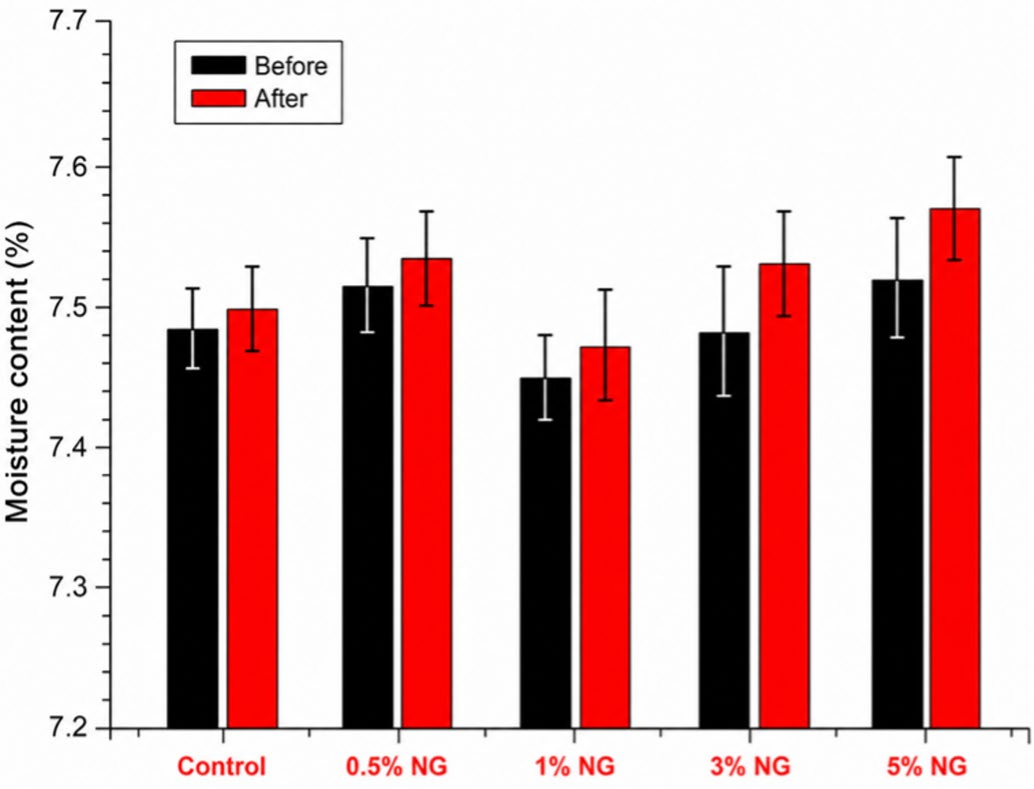
Moisture content (%) in different resin combinations used for printing.

The moisture content of NG-modified resins shows a consistent, albeit composition-dependent, variation compared with the control sample. The control resin exhibits a moisture content of 7.48% prior to heating and 7.49% post-exposure, indicating a marginal increase and suggesting minimal water uptake, as illustrated in Fig. 4. With the addition of NG (NG), the moisture content composition progressively increases across each formulation. Specifically, at 0.5% NG, the moisture content rises slightly from 7.51% (before) to 7.52% (after), indicating a minor change relative to the control. A similar trend is observed at 1% NG, where values increase from approximately 7.45–7.47%, although this composition exhibits the lowest overall moisture content among all samples.

A further increase in the concentration of the NG leads to a greater increase in the moisture absorption. There is an increase in the 3% NG sample from near 7.48% to around 7.52%, and in the 5% NG sample, the highest percentage is just 7.51–7.56%. Such an increase in moisture content in cases of increased NG loading is attributable to the increased surface area and the probable development of microvoids associated with the inclusion of nanofillers, which enhance moisture retention. Increased concentrations of NG are expected to cluster in specific areas, thereby enhancing their ability to trap moisture effectively. The consistent finding that all samples exhibit higher values after heat exposure supports the idea that heat prompts the release of moisture that was previously retained or absorbed in the resin matrix.

### Water absorption results

**Fig. 5:**
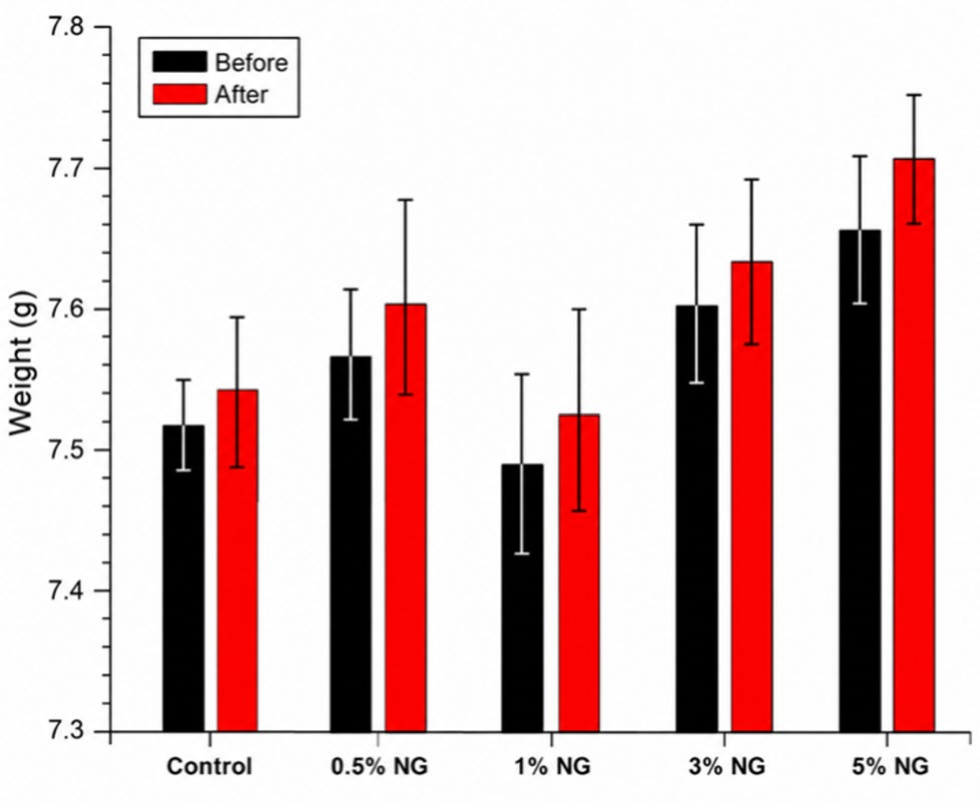
Water absorption (%) of different resin combinations used.

The absorption of water provides a clearer picture of how the resin reacts in a humid environment, particularly in the presence of NG. As shown in Fig. 5, the weight upon immersion increases slightly in all samples, indicating some water uptake regardless of composition. The control sample shows slight growth (from almost 7.51 g to almost 7.54 g), indicating that the water is relatively poor at absorbing the base resin. The trend is interesting with the introduction of NG. With 0.5% NG, the weight increase (7.56 g to 7.60 g) is slightly greater than that of the control, indicating that even a minor increase in filler can affect water absorption. But at 1% NG, the increase is relatively small (between about 7.49 g and 7.52 g) and suggests that this combination may provide a more compact, impermeable structure. This may be because NG dispersion improves at this level, thereby curbing water diffusion pathways.

With a further increase in NG content, the material absorbs more water. The sample with 3% NG shows an increase of about 7.60 g to about 7.63 g, whereas the rest of the samples with 5% NG show the largest increase of about 7.65 g to about 7.70 g.

The higher moisture content and water absorption at higher NG loadings are significant considerations for long-term dental applications. Saliva, temperature variations, and cyclic mechanical loads are constantly applied to restorative materials in the oral environment. Increased water absorption can lead to hydrolytic degradation of the polymer network, reduced structural stability, and dimensional changes, which can cause mechanical properties to deteriorate over time and reduce the filler–matrix interface strength. The increased water absorption of the 3 wt% and 5 wt% NG composites may be related to particle agglomeration and the presence of microvoids that allow moisture to permeate the resin matrix more easily. Among them, the moisture absorption of 1 wt% NG composite was comparatively low, indicating a compact, homogeneous microstructure likely to provide better long-term durability in oral conditions. These results also validate the choice of 1 wt% NG is the best level of reinforcement to balance mechanical characteristics and environmental stability.

### Gel content results

The gel content analysis revealed that all formulations exhibited a high gel content (> 99%), indicating the formation of a highly crosslinked and insoluble polymer network following UV curing. The control resin exhibited an average gel content of approximately 99.81%, confirming effective polymerization under the selected curing conditions. The incorporation of 0.5 wt% NG resulted in a comparable gel content of approximately 99.80%, suggesting that the addition of a small amount of nanographite did not adversely affect the curing process or network formation, as shown in Table 3. At higher NG loadings, a gradual decrease in gel content was observed, with average values of 99.53%, 99.38%, and 99.22% for the 1, 3, and 5 wt% NG composites, respectively. Although the differences are relatively small, this trend suggests that increasing nanographite concentration may slightly reduce curing efficiency. This behavior can be attributed to the strong UV absorption and light-scattering characteristics of nanographite, which reduce light penetration and consequently limit the degree of photopolymerization at higher filler contents. Nevertheless, the consistently high gel content across all formulations demonstrates that the SLA printing and post-curing conditions were sufficient to produce well-crosslinked polymer networks.

**Table 3:** Gel content of resin and NG composite.

Resin			0.5% NG		
Weight before curing (g)	Weight after curing (g)	Gel content %	Weight before curing (g)	Weight after curing (g)	Gel content %
6.30	6.29	99.84	6.09	6.08	99.84
6.39	6.37	99.69	5.69	5.67	99.65
6.24	6.22	99.68	6.16	6.15	99.84
6.25	6.24	99.84	5.99	5.98	99.83
6.27	6.27	100	5.86	5.85	99.83
Average		99.81			99.8

1% NG			3% NG			5% NG		
Weight before curing (g)	Weight after curing (g)	Gel content %	Weight before curing (g)	Weight after curing (g)	Gel content %	Weight before curing (g)	Weight after curing (g)	Gel content %
5.96	5.92	99.33	5.57	5.53	99.28	6.38	6.34	99.37
5.69	5.67	99.65	5.77	5.73	99.31	6.47	6.42	99.23
5.96	5.93	99.5	6.02	5.99	99.5	6.46	6.41	99.23
5.84	5.82	99.66	5.91	5.87	99.32	6.30	6.25	99.21
5.96	5.93	99.5	5.98	5.95	99.5	6.38	6.32	99.06
Average		99.53			99.38			99.22

At higher NG loadings, a slight reduction in gel content was observed. The average gel content decreased from 99.80% for the 0.5 wt% NG composite to 99.53%, 99.38%, and 99.22% for the 1, 3, and 5 wt% NG composites, respectively. Although the reduction is relatively small, it indicates that increasing NG loading may marginally reduce the curing efficiency of the UV-curable resin. This behavior can be attributed to the strong UV absorption and light-scattering characteristics of NG, which limit light penetration into the resin and reduce the extent of photopolymerization, particularly at higher filler concentrations. Increased NG loading promotes particle agglomeration, leading to localized shielding of the photoinitiator and the formation of non-uniform polymer networks. The slight variability observed in the individual measurements for the 3 wt% and 5 wt% NG composites are also consistent with the heterogeneous filler distribution and agglomeration observed in the optical microscopy and SEM analyses. All formulations exhibited gel contents greater than 99%, indicating that the applied SLA printing and post-curing conditions produced highly crosslinked, predominantly insoluble polymer networks despite a modest reduction in curing efficiency at higher NG concentrations.

### Tensile test results

**Fig. 6:**
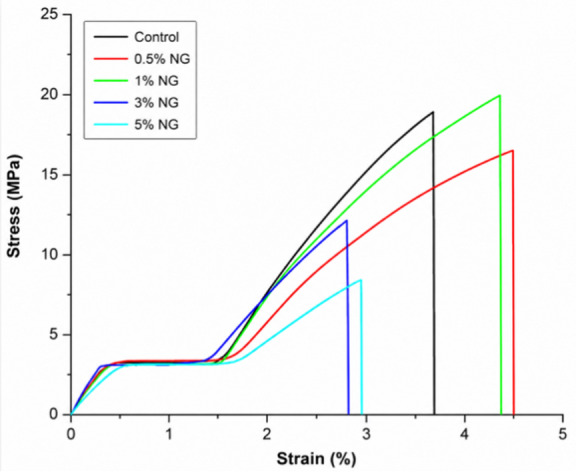
Tensile test results for different resin combinations.

The stress-strain behavior of the resin is evidently influenced by the NG loading, with the transition from strengthening to degradation being initiated by increased filler concentration. The maximum stress observed in the control sample is approximately 18 MPa at a strain of about 3.7–3.8, representing the baseline performance of the neat SLA resin, as depicted in Fig. 6. In the presence of 0.5% NG, the maximum stress slightly decreases to approximately 16 MPa, although the strain at failure increases to approximately 4.546. This indicates a minor reduction in load-bearing capacity and an increase in deformability, suggesting that at this low concentration, NG does not yet function efficiently as a reinforcement, potentially disrupting the polymer network. A significant enhancement is observed at 1% NG, with the composite exhibiting the highest stress of approximately 20 MPa and a high strain to failure (approximately 4.4–4.5). Such a combination of high strength and ductility suggests optimal dispersion and strong interfacial interactions between the NG platelets and the polymer matrix. NG is highly likely to transfer stress effectively at this concentration, as its layered structure facilitates better stress transfer. This is in line with previous microscopy and SEM results, in which the 1% NG sample had a more well-integrated and uniform morphology, with improved gel content and hardness. This is, however, an optimum loading, and above this loading, the mechanical performance is adversely affected. The NG sample (3%) has a lower peak stress of about 11–12 MPa and breaks earlier at a strain of 2.8-2.9, meaning it loses both strength and ductility.

Among all the formulations, the 5 wt% NG composites demonstrated the lowest tensile performance, exhibiting a maximum stress of approximately 7–8 MPa and a strain at failure of around 3.0%. These savings are significant, and strengths at 3% NG and above (5% NG and above) are reduced by 35-40% and more than 50%, respectively, relative to the control. This phenomenon could be explained by the agglomeration of NG particles at higher loadings, which leads to stress concentration areas, weak interfacial bonding, and perturbation of the curing process. This is evident in the SEM images, which showed voids, particle pull-outs, and heterogeneous areas in the high-NG samples; optical microscopy also indicated surface roughness and clustering.

The other crucial point is that all the samples had a similar initial response until approximately 1.5–1.8 strain, after which they split. The steeper increase in stress in the control and 1% NG samples indicates that the network is more effective at load-bearing under deformation. Conversely, the NG samples with higher values exhibit slower, earlier failure, suggesting that damage accumulation is prematurely manifested as structural defects. This also provided strong evidence that dispersion quality, not filler content alone, dictates mechanical performance. These results are consistent with the literature on polymer composites containing graphite and carbon. Research has demonstrated that NG platelets can improve mechanical properties at dilute concentrations due to their high aspect ratio and their ability to strengthen the matrix. Higher loadings decrease effective surface area and seriously undermine the composite by preventing restacking and agglomeration. For example, a study found that tensile properties are enhanced at low levels of graphite nanoplatelets, but aggregation causes a loss of properties at high loading levels^37^. Another study emphasized that it is of utmost importance that graphite fillers be uniformly dispersed, as this promotes reinforcement; poor dispersion leads to defects and poor mechanical performance^38^.

### Bending test results

**Fig. 7:**
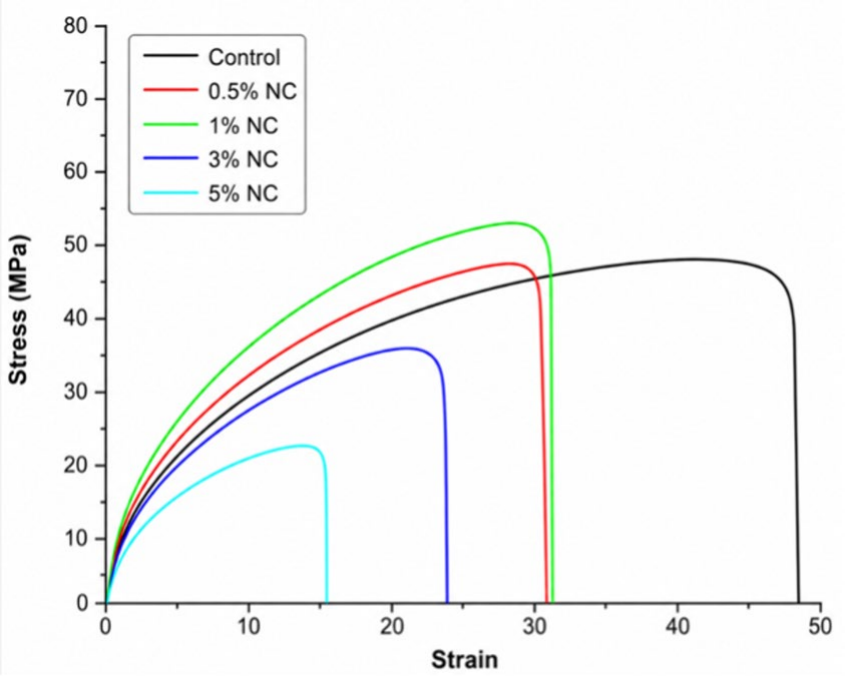
Bending stress–strain behavior of different resins.

The flexural characteristics of the resin and NG-reinforced composites exhibit a trend similar to the tensile outcomes and indicate a close relationship between filler dispersion and concentration and load-bearing performance, as shown in Fig. 7. The control sample has a maximum flexural stress of about 45 MPa at a strain of about 45.48, indicating excellent flexibility and consistent deformation during bending. The flexural stress is similar with the addition of the NG (approximately 0.5%), although the strain to failure is relatively lower (approximately 32–34), indicating that there is a small decrease in ductility, and yet the strength is not lost significantly. There is a noticeable improvement at 1% NG, where the composite attains the highest flexural stress of about 50 MPa, but failure occurs at a lower strain. This means that the resulting material is stiffer and less susceptible to bending deformation, presumably due to efficient load transfer between the polymeric hinge and the well-distributed hinges of the NG platelets. This is consistent with previous SEM and optical microscopy observations, which showed that the microstructure of the 1% NG sample was uniform and well-integrated, and with the improved tensile strength and gel content. But at a further higher stage of increasing the NG content, the flexural performance reduces.

The experiment with the 3% NG sample reveals a reduced maximum stress of about 32.33 MPa and a breakdown of about 26.27, indicating that the sample has lost both strength and flexibility. The 5 wt% NG composite showed the lowest flexural strength among all formulations, attaining a maximum stress of only 18–20 MPa before premature failure at strains of approximately 15–17, indicating a significant loss in structural integrity due to excessive nanographite loading. These deformations imply that an increase in NG levels leads to greater brittleness and reduced strength. The step-like character of the curves across all samples indicates that microcracks initiate and propagate progressively during bending. These steps are less abrupt in the control and 1% NG samples, which reveal steady crack growth and improved energy absorption. Conversely, the larger NG samples exhibit sharper failure behaviour, consistent with the presence of defects such as agglomerates and voids in SEM images. These flaws act as stress concentrators, accelerating crack formation and reducing the material’s capacity to resist bending forces.

Flexural results show that the best balance between flexural strength and stiffness is achieved with only 1% NG, with no compromise in deformation capability. Conversely, beyond NG loading (3–5%), it causes agglomeration, interfacial bonding, and early failure, thereby reducing bending performance. The trend is consistent with reported work on graphite- and graphene-reinforced polymer composites, in which good flexural properties are observed at both low and high concentrations; however, the properties deteriorate due to poor dispersion, leading to structural heterogeneity.

### Hardness results

**Fig. 8:**
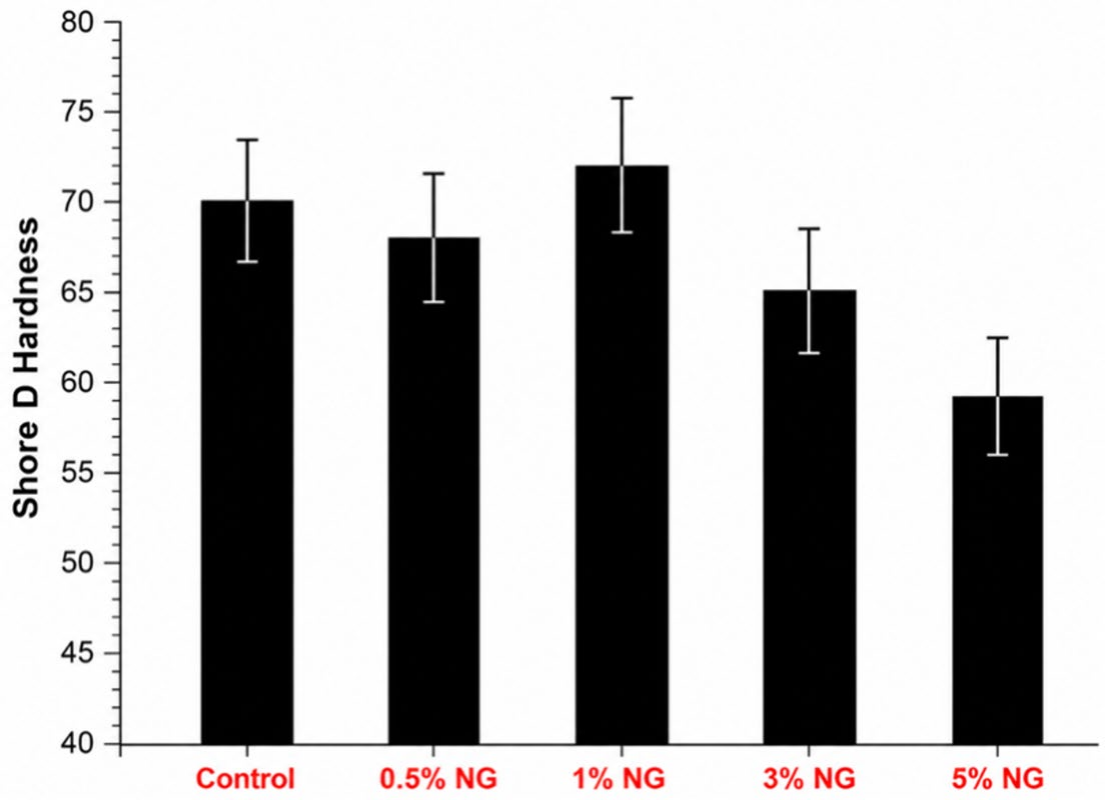
Shore D hardness values for different resin combinations used for printing.

The Shore D hardness data reveal a significant impact of NG concentration on the surface properties of SLA-printed resin. The control sample exhibits a hardness of approximately 70, which serves as a reference point, as shown in Fig. 8. Introducing 0.5% NG results in a slight decrease in hardness to about 68, likely due to minor disruptions in the polymer matrix at this low filler concentration, as seen in the figure. Interestingly, the hardness reaches a peak at a 1% NG concentration, with a value of around 72, exceeding that of the control sample. This indicates that, at this concentration, NG is effectively dispersed within the resin, enhancing load distribution and surface indentation resistance. Additionally, the improved crosslinking observed at this optimal concentration may further enhance the material’s strength. A noticeable decline in toughness is evident. The hardness value at 3% NG is approximately 65, but it drops significantly to about 59 at 5% NG. This decline at higher NG concentrations is likely due to particle agglomeration and the formation of microstructural discontinuities, which compromise the matrix’s overall stability and its resistance to deformation. Furthermore, excess filler can hinder effective curing, resulting in a network that lacks sufficient rigidity.

The results suggest that a minimal NG content (1–3%) positively affects surface hardness and may enhance wear resistance; however, at higher concentrations, the impact is adverse. In dental applications, where mechanical wear and surface resistance are crucial, it is important to achieve a balance by selecting an optimal filler content to ensure the desired equilibrium between reinforcement and structural integrity.

### Microscopic and microstructural investigation

**Fig. 9:**
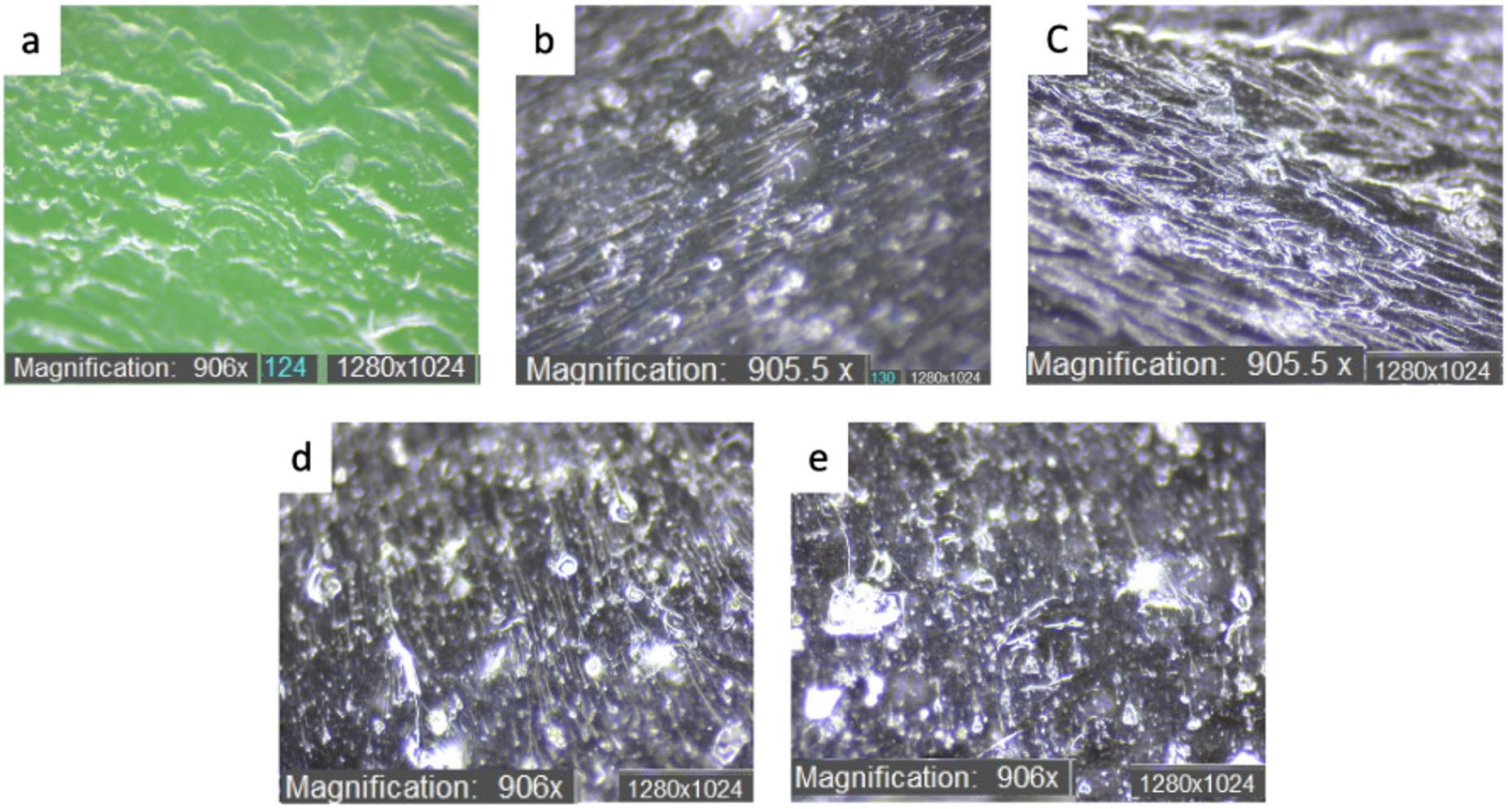
Optical microscopic surface for (**a**) Control (**b**) 0.5% NG (**c**) 1% NG (**d**) 3% NG (**e**) 5% NG.

The images from the optical microscopes provide a clear understanding of the development of the surface morphology of the SLA resin as the NG concentration increases. The control sample as Shown in Fig. 9a has a comparatively smooth, uniform surface with a homogeneous texture and few defects, indicating that the polymer matrix is well-formed and that defect occurrence is negligible. When 0.5% NG (b) is added, it starts to appear that the surface contains fine dispersed features, and there are minor textual differences indicating the initial distribution of NG in the matrix. The latter features are moderately dispersed, indicating no major clustering, and thus interfacial interactions are good at low filler loadings.

On the NG (c) surface, the morphology is more structured at a concentration of 1%, with visible alignment patterns and a slight increase in roughness. The nanofiller is more incorporated in the polymer network, resulting in a more interconnected morphology. This is in relation to better mechanical properties that have been found previously, since the dispersal is relatively homogeneous with no strong agglomeration. Nonetheless, when the content of NG is raised to 3% (d) the surface becomes significantly rougher and more of an uneven nature. There are separate bright spots and irregularities formed, which shows the development of filler-filled zones and begins the process of agglomeration of particles. These localized clusters cause the discontinuity of the uniformity of the matrix and add to the external irregularities. With the maximum loading of 5% NG (e), the surface is characterized by remarkable heterogeneity, bright spots, domains, and irregularities in microstructure. The structure is very irregular, agglomerative, and might have microvoids or defects. This implies that at elevated concentrations there is poor dispersion causing non-uniform stress distribution and a lack of structural integrity. Altogether, the findings of microscopy indicate a shift in a continuous and smooth surface in the control sample to the rougher and uneven morphologies, which increase as the NG content rises. Maximum dispersion and surface evenness is achieved at very low NG levels (0.51%) whereas greater loads include agglomeration and surface flaws which negatively affect the performance of the material, corroborating earlier studies^39^.

**Fig. 10:**
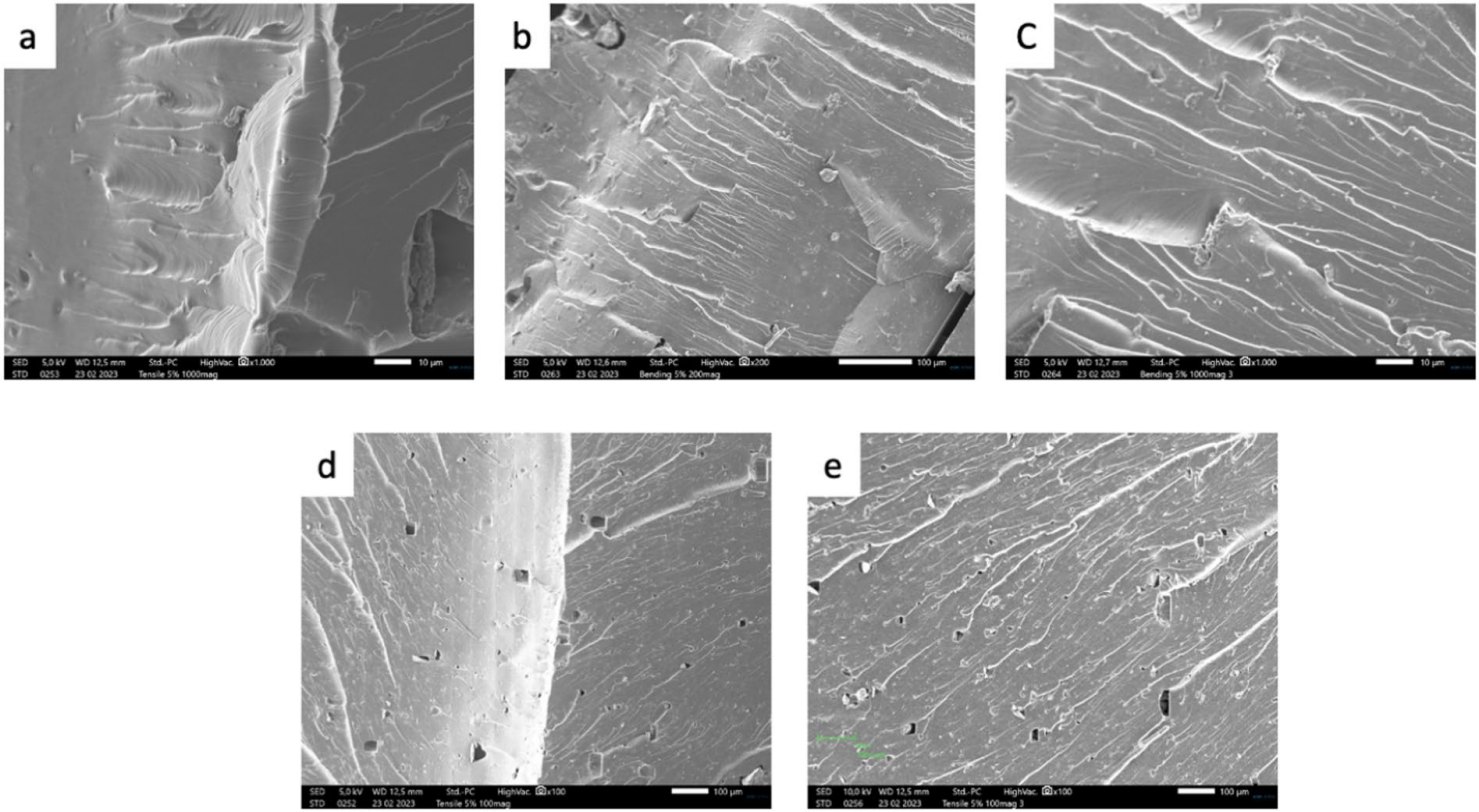
SEM images for (**a**) Control (**b**) 0.5% NG (**c**) 1% NG (**d**) 3% NG (**e**) 5% NG.

The SEM micrographs shown in Fig. 10, also corroborate the morphological trends in the optical microscopy analysis which is high-resolution evidence of the role of NG (NG) on the fracture surface properties of the SLA-printed resin. The control sample (a) has a comparatively smooth and continuous surface of fracture with apparent layer-shaped patterns corresponding to photopolymerized resin surfaces. The fact that there are no large voids or inclusions denotes a homogeneous polymer, which is in line with the homogeneous appearance under the optical microscope. The addition of 0.5% NG (b) causes the appearance of fine striations and some textural irregularities on the fracture surface which indicates the effective transfer of the stress between the matrix and the dispersed nanofillers. The NG seems uniformly distributed with no visible agglomerates that correlate with the higher hardness and middle-range gel content that was measured when the proportion of fillers was low. The surface appears more sharply outlined, with defined ridges on top and greater microstructural complexity at 1% NG (c). The morphology implies superior interfacial bonding and energy dissipation processes, which are traceable to greater crosslinking density and optimum filler dispersion, which is manifested by both gel content and mechanical performance.

When the NG levels are more intense, the SEM images have distinctive evidence of structural damage. Microvoids, particle pull-outs, and localized discontinuities are present in the 3% NG sample (d), indicating filler agglomeration and a lack of interfacial adhesion. These characteristics serve as points of stress concentration, which are associated with the decrease in hardness and the increase in moisture/water absorption mentioned above. The 5% NG sample (e) also exhibits more defects, larger voids, uneven cavities, and weakly bonded zones, which confirm severe agglomeration and a non-uniform distribution of NG in the matrix. These flaws disrupt the polymer network’s continuity and reduce its mechanical integrity. Previous research on carbon-based nanofillers in photopolymer systems has demonstrated a correlation between filler concentration and mechanical performance. Specifically, as the concentration of fillers decreases, mechanical performance improves due to enhanced dispersion and interfacial reactions. Conversely, increased filler concentration leads to agglomeration, reduced curing, and defect formation^40–42^. SEM analysis strongly correlates with optical microscopy, mechanical, and physicochemical findings, indicating that an optimal NG concentration of approximately 1% promotes an integrated and defect-minimized design. Any additional loading adversely affects the material’s structural integrity and functionality.

Although the present study demonstrates that 1 wt% NG provides the best balance of tensile, flexural, hardness, printability, and microstructural performance, the current work has some limitations. The study mainly focuses on initial mechanical, physicochemical, and morphological characterization. For dental applications, long-term functional properties such as wear resistance, fracture toughness, cyclic fatigue behavior, thermal aging, color stability, and biocompatibility were not evaluated. The resin employed in this work is not a clinically approved permanent dental restorative resin, but rather a model SLA-printable photopolymer resin. Thus, the results presented here can be viewed as a proof of concept for nanographite-reinforced SLA resin systems and are to be interpreted as a prerequisite for clinical application with dental-grade resin under oral conditions.

Dental restorative materials must simultaneously optimize several properties, such as mechanical strength, hardness, moisture resistance, wear resistance, fracture behavior, thermal stability, and biological response. In that regard, future studies could include multi-criteria decision-making (MCDM) approaches to aid more systematic material selection and ranking. The MCDM techniques have been widely used in the selection and optimization of dental restorative composites. The dental composites have been ranked several times by the R-method^43^, ENTROPY-VIKOR^44^, hybrid Entropy-VIKOR^45^, FAHP-TOPSIS^46^, FAHP-FTOPSIS^47^, and AHP-TOPSIS^48^. These methods can incorporate multiple material properties into a single model and be used to select the most effective formulation based on weighted performance criteria. Previous research has ranked, for instance, composites filled with nano-hydroxyapatite, ceramic particulate-reinforced composites, zirconia-reinforced composites, titanium oxide-reinforced composites, marble dust-reinforced composites, tricalcium phosphate-reinforced composites, and silica-reinforced composites as dental resin composites^49–51^. These studies illustrate the usefulness of MCDM tools for optimizing multiple conflicting properties simultaneously when choosing the most appropriate dental restorative composite. This will enable a more complete decision-making framework that takes mechanical, tribological, thermal, physicochemical, and biological performance into account, rather than focusing on a single property. This will reinforce the material selection process and enhance the clinical relevance of the developed dental composite system.

## Conclusion

The current study has shown that NG can be readily embedded in a UV-curable resin in SLA, making it suitable for dental use, but its performance will depend heavily on the filler concentration. In all the studies carried out, the findings consistently showed that 1% NG provided the best-balanced, technically favourable response. The resin exhibited the highest tensile strength, flexural stress, and Shore D hardness at this loading and retained a small, uniform microstructure. Optical microscopy and SEM also confirmed that the 1% NG composite showed better filler distribution and interfacial integration, and fewer visible defects, compared with the higher-loading samples. The following observations indicate that NG at the right concentration can be used as a reinforcing phase in the SLA resin, enhancing its capacity to transfer and share mechanical loads. This same tendency was supported by the physicochemical results. Low NG content was added to enhance UV absorption in the resin, which is necessary for photopolymerization during SLA printing. Viscosity was within the printable range across all formulations, indicating no adverse effects on resin processability with NG incorporation. Although the gel content increased with the NG concentration added, it did not necessarily correlate with improved performance at higher loadings. Indeed, the NG composites at 3% and 5% retained more moisture, absorbed more water, and exhibited textured surfaces and more noticeable agglomeration defects. These characteristics may accelerate hydrolytic degradation, weaken the filler–matrix interface, and reduce the long-term durability of the material in the moist oral environment, highlighting the importance of controlling nanographite loading for clinically relevant dental applications. These structural anomalies should have disrupted stress transfer in the matrix and favored premature failure under loading. This led to the loss of the mechanical benefits anticipated with increased filler content, as the microstructure became increasingly heterogeneous. An evident structure–property relationship emerged from the combined results. The addition of nanophase phosphorus in minute quantities, particularly the 1% increment, was found to reinforce the resin matrix without significant interference with curing or morphology. This optimum filler content, however, was overtaken when the filler content increased beyond this level; particle agglomeration, void formation, and interfacial weakness prevailed, resulting in poor tensile, flexural, and hardness performance. This implies that the efficiency of NG in SLA-printed dental composites cannot be improved solely by addition but is attained through an appropriate balance of reinforcement, dispersion, and curing efficiency. As far as applications are concerned, this is significant to the creation of next-generation additively manufactured dental materials. Dental resins should provide both printability and dimensional precision, adequate strength and surface integrity, and resistance to moisture-driven degradation in the oral cavity. According to the current study, the 1% NG-reinforced resin appears to be the most viable formulation, offering the best balance of printability, mechanical performance, and structural integrity. It should be noted that the present work employs a commercially available SLA-printable photopolymer as a model resin rather than a clinically certified dental restorative resin. Consequently, the findings should be interpreted as a proof-of-concept demonstrating the influence of nanographite reinforcement on SLA-printable resin systems. The current study showed that the addition of NG enhanced the mechanical, printability, and microstructural properties of UV-curable RCs processed by the SLA technique; however, several important factors associated with clinical dental applications were not included in this investigation. Specifically, wear resistance, fracture toughness, fatigue under cyclic loading, and biological performance (cytotoxicity and biocompatibility) were not assessed. To evaluate the long-term durability and clinical safety of dental restorative materials, these properties are necessary. Hence, future research efforts will involve detailed tribological, fracture mechanics, fatigue, and in vitro biocompatibility analyses based on the FDA and ISO guidelines for dental materials to further establish the potential of the developed nanocomposites for clinical dental applications. The controlled NG modification of UV-curable SLA resins is a promising candidate for provisional dental restorations, temporary prosthetic components, dental models, and other non-load-bearing dental applications.

## Data Availability

Data will be made available on request.
